# Disease-modifying agents in multiple sclerosis

**DOI:** 10.4103/0972-2327.58280

**Published:** 2009

**Authors:** P. K. Coyle

**Affiliations:** Department of Neurology, Stony Brook University Medical Center, Stony Brook, New York, USA

**Keywords:** Disease-modifying agents, glatiramer acetate, interferon beta, multiple sclerosis therapy, natalizumab

## Abstract

Since 1993, six disease-modifying therapies for multiple sclerosis (MS) have been proven to be of benefit in rigorous phase III clinical trials. Other agents are also available and are used to treat MS, but definitive data on their efficacy is lacking. Currently, disease-modifying therapy is used for relapsing forms of MS. This includes clinically isolated syndrome/first-attack high-risk patients, relapsing patients, secondary progressive patients who are still experiencing relapses, and progressive relapsing patients. The choice of agent depends upon drug factors (including affordability, availability, convenience, efficacy, and side effects), disease factors (including clinical and neuroimaging prognostic indicators), and patient factors (including comorbidities, lifestyle, and personal preference). This review will discuss the disease-modifying agents used currently in MS, as well as available alternative agents.

## Introduction

Multiple sclerosis (MS) remained an untreatable disease until 1993, when the first disease-modifying agent became available.[1–3] Since then five more disease-modifying agents have come into use.[4–11] The MS disease-modifying agents have multiple benefits [Table 1]. Current opinion favors early use for best long-term results. Since, in MS, there is accumulating permanent damage, even when patients appear stable, early intervention makes sense. Although the disease-modifying therapies have not been available for very long, they already are reported to decrease late disability as well as transition from relapsing to secondary progressive MS.[12,13]

**Table 1:** Benefits of MS disease-modifying agents

Relapses Lower relapse rate Milder relapses Higher proportion of relapse-free patients Longer time to next relapse Neurologic examination Less development of disability/sustained worsening Quality of life Maintained or improved Magnetic resonance imaging Decrease in T2, T1, and contrast lesion development Lesion burden stabilized or decreased Less development of CNS atrophy

## Eligible Patients

The disease-modifying agents are used for the relapsing forms of MS. This is predominantly clinically isolated syndrome (CIS)/first-attack high-risk patients and relapsing MS patients, but also includes secondary progressive (SP) and progressive relapsing MS patients experiencing clinical attacks superimposed on their slowly worsening course. Other markers for relapsing disease are contrast lesion activity on magnetic resonance imaging (MRI) and rapid clinical worsening. None of the currently available disease-modifying agents have been proven to be of benefit in progressive MS.

## Established Agents

Six agents have been proven in phase III randomized, prospective, placebo-controlled trials to benefit MS [Table 2]. They all affect the immune system. Onset of action appears to be fairly rapid (within 12 weeks). Four [the interferon-betas (IFNßs) and glatiramer acetate (GA)] are considered first-line agents, while two (natalizumab and mitoxantrone) are considered second-line agents as they have more significant risk of side effects. All the disease-modifying agents (with the exception of mitoxantrone) are very expensive; annual costs run well over US $20,000. They require parenteral administration: either subcutaneous (SQ) or intramuscular (IM) injection or intravenous (IV) infusion.

**Table 2:** Disease Modifying Agents for multiple sclerosis

Agent	Class	Dose	MOA	Comments
First line				

IFNß1b (Betaseron^®^, Betaferon^®^, Extavia^®^	Anti inflammatory/regulatory cytokine	250 μg SC every other day	Decreases matrix metalloproteinases, adhesion molecules, T cell activation increases suppressor activity, autoreactive T cell apoptosis	First approved agent requires laboratory testing indefinitely can generate Nabs (<35%) Category C pregnancy drug
IFNß1a (Aovonex^®^)	See above	30 ug IM weekly	See above	Requires laboratory testing the first year Category C pregnancy drug can generate Nabs (<5%) Category C pregnancy drug
IFNß1a (Rebif^®^, new formulation rebif,^®^	See above	44 (22) μg SC thrice weekly	See above	Requires laboratory testing indefinitely Can generate NAs (20%) Category C pregnancy drug
GA (Copazone)	Amino acid polymer	20 mg SC daily	Generates anti inflammatory regulatory cells. Th1 to Th2 switch, increases BDNF	No laboratory testing required Category B pregnancy drug

Second line				
Natalizumab (Tysabri)	Anti adhesion molecule	300 mg iv monthly	Blocks cell penetration in to target cell body organ	Serious risk, involves PML (as high as 1 in 1000)
	Monoclonal antibody			Can generate Nabs (6%) Category C pregnancy drug
Mitoxantrone (Novantrone), Cnedione)	Anthracenedione	12mg/m^2^ IV every 3 months (max 140 mg/m^2^)	Intercalates in to DNA blocks DNA repair	Serious risks, involves cardiomyopathy, treatment related leukemia, infertility, Category D Pregnancy drug

MOA= Mechanism of action; PML = Progressive Multifocal Leukoencephalopathy

## IFNß

IFNß is a type I anti-inflammatory/regulatory IFN. It occurs naturally in the body, but the IFNß used for treatment is produced by recombinant technology and is administered at high levels. IFNß was initially used in MS because of its antiviral action but is now believed to be of benefit in MS due to its immunomodulatory properties.[14] It acts systemically to decrease T cell activation, enhance suppressor cell activity, and inhibit proinflammatory cytokines. It also stabilizes the blood–brain barrier and shuts down MRI enhancement. IFNß can provoke detectable antibodies, both binding (BAbs) and neutralizing (NAbs). All NAb-positive patients have BAbs, so they are often used as a screening assay. Some patients have sustained high-titers of NAbs, which interferes with IFNß efficacy.[15,16] However, only a minority of patients appear to fall into this category. It is very rare to become NAb positive after 2 years. Of those who develop NAbs, many revert spontaneously to permanent seronegativity. NAb assays are available, but routine testing is controversial.[15,16] Assays which detect loss of ability to induce MxA protein, an IFN-specific product, may be a better marker of IFNβ ineffectiveness.[17]

Major IFNß side effects include flu-like reactions in the first 12 weeks after initiation of therapy.[18,19] This can be eliminated in most patients by a dose-escalation schedule, premedication with anti-inflammatory agents (such as ibuprofen or naproxen), and early evening dosing (so that peak levels are achieved during sleep). IFNβ can produce abnormalities in white blood cell, red blood cell, and platelet counts, as well as in liver enzymes. These are typically transient but are sometimes pronounced enough to require reduction or even discontinuation of IFNβ. Thyroid function abnormalities may also occur. Liver enzymes, complete blood count (CBC), and thyroid function tests are routinely monitored during therapy: CBC and liver enzymes every 3 months during the first year of therapy and then twice a year, and thyroid stimulating hormone once a year. This is the standard monitoring schedule for the higher, more frequently dosed IFNßs; however, with once-a-week IFNβ many discontinue monitoring after the first year. Pre-existing liver disease is a relative contraindication for IFNβ treatment. Some patients report worsening of headache and episodic depression with IFNß. Finally, the IFNßs given by SC injection are associated with variable degrees of injection site reactions, ranging from reddening of the skin (which is almost universal) to pain, induration and, rarely, even skin breakdown. Appropriate injection technique and rotation of sites are critical; in addition, certain maneuvers may decrease injection site reactions (for example, preheating of the skin, injecting body temperature drug, changing needle depth/angle, etc).

### IFNβ1b

IFNß1b (Betaferon^®^, Betaseron^®^) was the first MS disease-modifying agent.[1–3] IFNß1b has three molecular differences from human IFNß: it is not glycosylated [since it is made in bacteria (*Escherichia coli*)], is missing the N terminal methionine, and has a serine for cysteine substitution at position 17. IFNß1b has 165 amino acids, with a molecular weight of 18.5 kilodaltons (kD). The lyophilized protein product (0.3 mg, or 8 million international units) is reconstituted with supplied diluent and injected at a dose of 250 μg SC every other day. There have been four placebo-controlled phase III trials with IFNß1b: the original pivotal trial in relapsing MS,[1–3] two trials in SP MS (from Europe and North America)[20,21] and one trial in CIS/first-attack high-risk MS.[22] These studies found that IFNß1b significantly reduced relapse rate (both annualized relapse rate and relapse frequency per patient), increased the proportion of relapse-free patients, decreased moderate to severe relapses, and decreased brain MRI lesion burden in relapsing MS. Relapse and MRI benefits were confirmed in the SP MS studies, but decreased progression was only found in the European SP MS study. Subsequent analysis indicated that these patients were closer to the relapsing phase than the North American SP MS study patients, suggesting that IFNß1b had not demonstrated a benefit on true progression.[23] In the CIS study, IFNß1b *vs* placebo significantly decreased subsequent relapses and MRI lesion activity, defining either clinically definite MS or MS meeting the International Panel McDonald Criteria.[24] Analysis at 3 and 5 years indicated that immediate *vs* delayed treatment of CIS was associated with less late disability as well as better cognitive function as measured by the PASAT, a cognitive processing speed test.[25,26]

Recently, a true bioidentical IFNß1b (Extavia^®^) was introduced in the market in Europe and North America. This alternative IFNβ1b has been offered at reduced cost in order to gain market share.

### IFNß1a (IM)

IM IFNß1a (Avonex^®^) has been available since 1996. Its amino acid sequence is identical to that of human IFNß. It is glycosylated, although not necessarily in the same pattern as natural IFNβ. IFNβ1a is injected at a dose of 30 μg IM once a week; it comes as a prefilled syringe preparation or as a powdered form that is reconstituted before use. It has 166 amino acids and a molecular weight of 25.5 kD. It is produced in Chinese hamster ovary cells. IM IFNß1a has been studied in two phase III trials, one (MSCRG) in relapsing MS and the other (CHAMPS) in CIS patients.[5,27] In the pivotal relapsing MS trial, patients treated with IM IFNß1a showed significantly less disability than placebo as measured by sustained expanded disability status scale (EDSS) worsening, relapses, and contrast MRI lesion number and volume. In the CHAMPS CIS trial, IM IFNß1a significantly reduced occurrence of relapses (defining clinically definite MS) as well as new MRI lesion activity.[27] IM IFNß1a was also studied at double dose (60 μg IM weekly) in a phase III SP MS (IMPACT) trial.[28] Although there was a significant effect compared to placebo on worsening based on the MS Functional Composite (25-foot timed walk, 9-hole peg test, and PASAT), there was no effect on EDSS. Ultimately, the IMPACT trial was not accepted by regulatory groups as having documented a benefit in SP MS.

### IFNβ1a (SC)

SC IFNβ1a (Rebif^®^) is available in a prefilled syringe, either 22 μg or 44 μg in 0.5 ml volume. An escalating dose starter kit is also provided. It is given by SC injection three times a week (generally Monday, Wednesday, and Friday). In the United States, use is virtually confined to the 44 μg dose. The pivotal relapsing (PRISMS) trial evaluated 22 and 44 μg of SC IFNβ1a *vs* placebo. At 2 years, both doses showed significant ability to decrease relapses, sustained disability, and MRI lesion activity.[6,7] The only difference between the two doses was that the 44 μg dose had a superior effect on decreasing T2 MRI lesion burden. In the extension PRISMS study, in which 80% of the original cohort participated, the placebo group was re-randomized to 22 or 44 μg doses. The best outcome at 4 years was noted in the group originally randomized to the 44 μg dose.[29]

SC IFNβ1a was also studied in CIS patients (the ETOMS trial).[30] The dose used was very low (22 μg SC weekly *vs* placebo). Nevertheless, there was a significant benefit in decreasing both relapses and MRI lesion activity. An ongoing European CIS study is evaluating 44 μg given either once or three times a week *vs* placebo.

The phase III SP MS (SPECTRIMS) trial evaluated 22 and 44 μg *vs* placebo and basically showed results identical to that seen in the North American IFNβ1b SP MS trial – i.e., no effect on EDSS progression, but a beneficial effect on relapses and MRI activity.[31]

Recently, a new formulation product, free of fetal bovine serum and human serum albumin, has replaced SC IFNβ1a in Europe and Canada and is expected to be approved for use in the United States. It has the advantage of having less injection site reactions.

## Glatiramer Acetate (GA)

GA (Copaxone^®^) is made up of the acetate salts of synthetic polypeptides (average molecular weight 5–9 KD), consisting of four amino acids found in myelin basic protein (MBP): L-glutamic acid, L-lysine, L-alanine, and L-tyrosine. It is given at 20 mg SC daily byprefilled syringe. A biophysical analogue of MBP, it is believed to work by generating GA-responsive regulatory cells that may migrate into the CNS to downregulate immune responses and subsequent damage. It also promotes a shift from Th1 to Th2 cells. Unlike IFNβ, GA does not appear to affect the blood–brain barrier. There have been three trials in relapsing MS that have supported its use. The earliest one was a very small single-center double-blind study that involved 50 patients.[32] GA *vs* placebo treatment reduced relapses over 2 years. The second multicenter trial entered 251 patients and also showed a significant benefit in reducing relapses.[4] However, these studies did not evaluate neuroimaging. A third trial focused on MRI and entered patients with baseline contrast lesion activity.[33] Over 9 months, MRI lesion activity was significantly suppressed with GA treatment. GA has also been studied in 481 CIS patients (PreCISE trial), with significant delay in subsequent relapses and MRI lesion activity.[34] GA was evaluated in the only completed phase III trial to date for primary progressive MS, the PROMISE trial.[35] This study was stopped early because a progression effect could not be met at year 3. However, there was a trend toward a treatment effect compared to placebo and, in a *post hoc* analysis, there was a significant treatment effect in men with PPMS.

A recent (FORTE) trial found no significant difference between 20 *vs* 40 mg SC of GA daily for relapsing MS.

The major side effects of GA include injection site reactions. Occasionally these can be quite bothersome, causing pruritus, fibrotic nodules, or lipoatrophy with skin dimpling. Some 10–15% of patients experience one or more episodes of a systemic reaction/immediate postinjection reaction. This is benign and transient, but it can be frightening. It involves chest pain, palpitations, anxiety, difficulty in breathing, and flushing, and is similar to a severe panic attack. Urticaria and lymphadenopathy can occasionally be problems. No blood monitoring is needed with GA. BAbs are seen in all cases, but NAbs do not seem to occur.

### Natalizumab

Natalizumab (Tysabri^®^) is a humanized monoclonal antibody directed against α 4-integrin, an adhesion molecule expressed on all leukocytes except neutrophils. It is given every 4 weeks at a dose of 300 mg as an IV infusion over 1 h. Natalizumab blocks cell migration into a targeted body organ (in the case of MS, the CNS) by preventing cell adhesion to endothelium. Because it also binds to osteopontin and fibronectin, it may have the additional effects of blocking T cell activation and cell retention within the CNS. Natalizumab was evaluated in two phase III relapsing MS trials. The monotherapy AFFIRM trial compared natalizumab to placebo, while the combination SENTINEL trial added natalizumab or placebo to patients already on IM IFNβ1a who had experienced one or more breakthrough relapses.[10,11] At 2 years, patients treated with natalizumab showed significantly better suppression of relapses, disability, and MRI lesion activity.

Natalizumab is very well tolerated. The major side effects associated with infusion are headache and fatigue, increase in infections (respiratory and urinary tract), and gastrointestinal upset. Hypersensitivity reactions (less than 1% of patients develop anaphylaxis) include urticaria, dizziness, flushing, chest pain, and hypotension, and occur within 2 h of infusion. Persistent NAbs occur in 6% of patients and are associated with increased risk for infusion hypersensitivity reactions as well as complete loss of efficacy. Persistent NAbs should lead to discontinuation of therapy. Virtually all NAb positivity is present by 6 months, but positive results should be confirmed at 3 months to check that NAbs are persistent.

The major concern with natalizumab is the development of progressive multifocal leukoencephalopathy (PML). In the original study cohort of 3,000 subjects, PML developed in three cases (two MS patients from the SENTINEL trial and one Crohn patient). This led to a voluntary withdrawal of the drug from the marketplace for over a year before it was reintroduced in late June 2006. In the United States, before starting treatment, all patients sign a consent form accepting the 1 in 1000 risk of PML. Baseline brain MRI is performed. Patients are prescribed the drug for 6 months at a time and are asked a set of questions just prior to every infusion to screen for PML symptoms. As of November 2009, there have been 27 postmarketing cases of PML (including 4 deaths), with natalizumab monotherapy in over 63,000 treated cases. Interestingly, 17 are from Europe and 10 from the United States. Half had prior immunosuppressive therapy. The current approach to the use of natalizumab is presented in Table 3.

**Table 3:** Current approaches to use of natalizumab

Patient selection -Relapsing forms of MS -Generally patients who have failed one or more agents -Can be used first-line in selected treatment-naïve patients (very active disease; African Americans; those unable to tolerate injections) -Potential concerns when used in patients with pre-existing liver disease disease or prior immunosuppressive therapy -Not used in immunocompromised MS patients (active viral hepatitis, HIV seropositivity, etc.) Other issues -Used only as monotherapy -PML risk 1 in 1,000 at 24 to 36 months; 27 postmarketing cases in over 63,000 treated as of November 2009 -No MS PML cases thus far with <12 months therapy -washout period not indicated for IFNß, GA, glucocorticoid therapies; washout period recommended for immune suppressive therapies -Baseline brain MRI ± contrast prior to initiation of natalizumab -No mandatory routine monitoring; can do liver enzymes at 1-3 months, NAbs at 1-3 months; NAbs at 6 months or with infusion reactions/relapse; surveillance MRI every 12 months -Suspicion of PML should trigger stopping therapy, brain MRI ± contrast; if suggestive, proceed to lumbar puncture for JC virus PCR; if positive proceed to plasma exchange (5 exchanges over 10 days) -PML risk 1 in 1,000 at 24 to 36 months; 24 postmarketing cases (4 deaths) in 60,700 treated as of october 2009

It has been suggested that non-Caucasians (African Americans and Hispanics) respond just as well to natalizumab as do Caucasians. This is in contrast to the less effective IFNβ response of African Americans and has led some experts to consider natalizumab as the preferred treatment in African Americans with relapsing MS, especially since they have a worse prognosis to begin with.[36]

### Mitoxantrone

Mitoxantrone (Novantrone^®^) is an antineoplastic anthracenedione. It intercalates into DNA, causing cross-links and strand breaks. It is cytotoxic for both proliferating and nonproliferating cells and inhibits B cells, T cells, and monocytes/macrophages. Mitoxantrone is given at dose of 12 mg/m^2^ by IV infusion every 3 months. The lifetime maximum dose in MS is 140 mg/m^2^ (approximately 11 doses). This limit reflects concerns about accumulating cardiomyopathy risk. Mitoxantrone is a second-line agent because it carries risk for significant adverse effects. These include cardiomyopathy, treatment-related leukemia, leukopenia, and infection, and menstrual disorders/infertility. It is generally used in patients who have failed first-line treatments. In recent trials, short-term mitoxantrone (for 3–6 months) has been used as induction therapy, to be followed by first-line therapy.

In the United States, use of mitoxantrone in MS requires evaluation of ejection fraction (by MUGA scan or 2D echocardiogram) before each dose, and then annually for the lifetime of the patient. Therapy is discontinued if the ejection fraction falls significantly or is <50%. Treatment-related acute myelogenous leukemia (AML) is reported in 0.25% of treated patients, typically within 5 years.

The risk of AML is greater in patients who have received other cytotoxic agents. Blood counts and liver enzymes are checked before each mitoxantrone dosing. Infertility is chiefly a risk for women over age 35.

The major mitoxantrone (MIMS) trial entered relapsing and SP MS cases, randomized to one of three arms: placebo, and 5 mg/m^2^ or 12 mg/m^2^ of mitoxantrone for 2 years.[8,9] The 12 mg/m^2^ dose showed the clearest benefit on relapse, disability, and MRI parameters. A second smaller study evaluated rapidly worsening patients randomized over 6 months to monthly pulse methylprednisolone, or mitoxantrone plus methylprednisolone.[37] Again, the mitoxantrone group showed significant relapse, disability, and MRI benefits.

Sometimes the dosing of mitoxantrone is personalized. It has been used monthly as well as every 6 months and has been used at the lower (5 mg/m^2^) dose. With the availability of multiple options, mitoxantrone use has diminished.

## Head-to-head trials

The only definitive way to compare efficacy between agents is to study them within the same trial. There are very few randomized, prospective head-to-head trials between the available disease-modifying agents [Table 4]. Two such trials (EVIDENCE and INCOMIN) suggested that higher, more frequently dosed IFNβs may have greater efficacy than once-a-week IFNβs.[38–41] Two other very recent trials (REGARD and BEYOND) that compared the high-dose and frequently dosed IFNβs with GA found very similar times to onset of action and equal ability to suppress relapses.[42,43] Most remarkably, these two trials found that the IFNβs and GA were associated with approximately one relapse on treatment every 3 years. This is much better than the results reported from the IFNβ and GA pivotal trials a decade or more earlier. It suggests that modern relapsing MS trials are entering patients with earlier stage of disease and less active disease, and these patients may respond better. Demographics for the most part confirm overall shorter disease durations and lower average EDSS scores.

**Table 4:** Head-to-head trials

Trial	Agents	Demographics	Outcome	Comments
Incomin	SC IFNß1b 250 μg every other day vs IM IFNß1a 30 μg weekly	n = 188 Relapsing MS; 2 year trial	Proportion relapse free, relapse rate, disability, New T2 MRI lesions significantly better with SC IFNß1b vs IM IFNß1a	Blinded for MRI but not treating physician Study supports greater efficacy for higher, more frequently dosed IFNß
Evidence	SC IFNß1a 44 mcg 3 × weekly vs IM IFNß1a 30 μg weekly	n = 677 Relapsing MS; 24- and 48- week trial, with voluntary extension SC IFN1a crossover to 64 weeks	Proportion relapse free, active MRI lesions significantly better at 24 and 48 weeks with SC IFNß1a vs IM IFNß1a; crossover showed better relapse reduction, fewer T2 active lesions in IM to SC IFNß1a switchers	Study supports greater efficacy for higher, more frequently dosed IFNß
Regard	SC IFNß1a 44 mcg 3 × weekly vs SC GA 20 mg daily	n = 764 relapsing MS; 96 week trial	No difference in time to first relapse	On treatment relapses much fewer than expected ARR much lower than in pivotal trials for SC IFNß1a (0.3), GA (0.29)
Beyond	SC IFNß1b 250 μg vs 500 μg every other day vs SC GA 20 mg daily	n = 2,244 Relapsing MS; 96 week trial	No difference in relapse rate	ARR much lower than in pivotal trials for SC IFNB1b (0.35), GA (0.34); also low in 500 mcg IFNß1b (0.34)

ARR = Annualized relapse rate

## Other agents

There are a number of other agents that are still used to treat MS, even though they have not been established for use by phase III trials. These can be divided into immunosuppressant and immunomodulatory approaches [Table 5]. The rationale for general nonspecific immune suppression is based on the theory that MS involves an immune-mediated damage process.

**Table 5:** Other agents used to treat MS

Immunosuppression
Azathioprine Bone marrow suppression Cyclophosphamide Methotrexate Mycophenolate mofetil Pulse glucocorticoids
Immune modulation
IV immunoglobulin Plasma exchange

### Immunosuppression

#### Azathioprine:

This is a purine antagonist which affects DNA replication. It affects T cells more than B cells and is considered to have a better safety profile than cyclophosphamide or methotrexate. It has been a particularly popular drug in Europe for the treatment of MS and was widely used through the early 1990s. It was actually approved for use in Germany for treatment of MS. A meta-analysis of seven controlled trials found a modest benefit on EDSS disability for relapsing and SP MS. A recent Cochrane analysis reviewed five trials (*n* = 698 patients) and concluded that there was a benefit with regard to reducing relapses.[44] Azathioprine is used orally at 50–200 mg daily to lower the WBC count. It has also been used in combination with IFNß to control breakthrough disease activity in small series.[45] It is very well tolerated, with gastrointestinal effects and leukopenia being the major side effects followed by infections, anemia, thrombocytopenia, and allergic reactions.[46] Treatment duration of over 10 years and cumulative doses of over 600 gm may increase the risk of later malignancy.

#### Bone marrow transplantation:

Immunoablative therapy has been proposed as a possible cure for immune-mediated diseases such as MS. The concept is to reset immunological memory directed against autoantigens. In fact, autologous hematopoietic stem cell transplantation has been evaluated in several hundred refractory MS patients.[47,48] Currently, bone marrow transplantation is an unproven treatment and should only be performed in the context of clinical trials. It involves several stages. First, stem cells from the host are harvested. This avoids an allogeneic source and removes the risk of graft *vs* host disease. Second, the patient undergoes intensive therapy to ablate his/her entire (myeloablative) immune system or selected components (nonmyeloablative). Finally, the purified stem cells are transplanted to reconstitute a naïve, healthy, new immune system. Bone marrow transplantation carries a mortality rate of 2–3% in the most recent reports, and a higher morbidity rate. More intensive ablative regimens have greater toxicity. Individual patients however, have been reported to stabilize, or even show reversible improvement, for at least several years. Ideal candidates would be earlier in the disease process and still ambulatory, without severe permanent damage. However, it is difficult to justify this treatment except in clearly worsening patients who have failed proven therapies. Bone marrow transplantation may slow down CNS atrophy but does not appear to stop it. It does not fully ablate the CNS inflammatory process.[48,49]

#### Cyclophosphamide:

Cyclophosphamide (Cytoxan^®^) is an alkylating agent which binds to DNA, and interferes with DNA and RNA synthesis. It affects both B and T cells, modulates the cytokine production network in MS, and crosses the blood–brain barrier. It has been used principally in rapidly deteriorating relapsing MS and early progressive MS. Cyclophosphamide is most often given as IV pulses.[50] It can be used as induction therapy, induction followed by pulse maintenance therapy, or pulse maintenance alone. Often it is given initially monthly and then later spaced out over up to 6 months. For such therapy, the lifetime exposure is limited to the 80–100 gm range. Cyclophosphamide dose is generally in the range of 750–1,000 mg/m^2^ to decrease the WBC count to as low as 2,000. IV methylprednisolone is often added and may enhance the cyclophosphamide effect.

The MS subset most likely to respond to cyclophosphamide are the younger patients (18–40 years of age), with shorter disease duration (fewer years into the progressive phase), superimposed relapses (one or more in the prior year), contrast lesion activity, and rapid worsening disease.

Several recent studies have reported one-time use of high-dose cyclophosphamide for severe, refractory, immune-mediated diseases such as MS.[51–53] Patients are hospitalized for about 2 weeks. They are given 50 mg/kg cyclophosphamide IV daily for 4 days (total 200 mg/kg). Patients may need to be treated for low WBC, RBC, and platelet counts and may need to be given routine prophylactic antimicrobials. In small series, both active relapsing and SP MS patients were reported to stabilize over a follow-up of up to 2 years.

The major side effects of cyclophosphamide therapy include nausea, hair loss, leukopenia with infections, menstrual disorders/infertility, bladder toxicity, and late malignancy. Patients are premedicated with antiemetics; MESNA and excellent hydration are used to minimize the risk of hemorrhagic cystitis; annual urine cytology screens are performed while on treatment, and yearly cystoscopy is performed after 3 years of treatment.

#### Methotrexate:

Methotrexate is an antimetabolite agent that also suppresses chemokine expression (CXCR3 and CCR4) and inflammatory cytokine production. Its use in MS has largely involved oral doses ranging from 7.5–20 mg weekly, either as monotherapy or as combination therapy with a first line disease-modifying agent.[54–59] It has also been used IV at high dose, with activated folic aid (citrovorum) rescue therapy.[60] Monotherapy has generally been used for progressive MS. Therapy should involve daily folic acid.

#### Mycophenolate mofetil:

Mycophenolate mofetil (CellCept^®^) is an oral antimetabolite, a selective inhibitor of inosine 5'-monophosphate dehydrogenase type II, the enzyme that synthesizes guanine, the purine nucleotide, in - activated B and T cells and macrophages. There is limited data on its use in MS.[61–63] The daily dose is 1 gm twice a day given on an empty stomach. Adverse effects include gastrointestinal upset, sleep disturbances, headache, tremor, dizziness, and muscle/joint pain.

#### Pulse glucocorticoids:

Glucocorticoids are the standard treatment for significant MS relapses. Patients are told that their use may promote a more rapid recovery, although the ultimate degree of recovery is unaffected. The most common regimen involves 1 gm of IV methylprednisolone given daily over 30 min, for 3–5 (up to 7) days. This regimen can be used to treat the rare relapse during pregnancy, although it is preferable to avoid treatment in the first trimester (during organogenesis) if possible. It is becoming uncommon to use an oral taper; if needed, the high-dose regimen is repeated/prolonged. It appears that the benefits are conveyed by the high dose rather than the route of administration. High-dose oral treatment seems to work just as well as IV, since bioavailability is excellent.[64–66] The mechanism of action is believed to reflect anti-inflammatory and anti-edema properties, including decrease in adhesion molecule production, proinflammatory cytokines, and circulating CD4+ T cells and B cells.

There are limited studies which suggest that regular pulsed glucocorticoids may have disease-modifying benefits.[67,68] There is no universally accepted regimen and, again, 1 gm of IV methylprednisolone given on 1 day every month or for 3 days every 2 months are the most common protocols. Patients should show some discernable benefit, to justify continuation of this therapy. Several small studies have added pulse steroids in combination with a proven disease-modifying agent, but this remains an unproven treatment regimen.[58,69]

The adverse effects of glucocorticoids are well known, and relate especially to chronic high-dose use. They include weight gain/edema, sleep and mood disturbance, gastrointestinal upset, hyperglycemia, bone changes, striae, infection, cataracts, myopathy, and avascular necrosis of the femoral head.

### Immune modulation

#### IV immunoglobulin:

IV immunoglobulin is accepted therapy for several peripheral nervous system immune-mediated disorders, but it has not been documented convincingly to benefit MS. A phase III trial in secondary progressive MS was negative.[70] Phase II trials in relapsing MS have shown mixed results, although a meta-analysis concluded a probable benefit on relapse and MRI parameters.[71] A single-center study of CIS/first-attack high-risk patients did suggest a benefit over placebo, and there are multiple small-scale studies that have suggested a postpartum benefit. One postpartum regimen involves 60 gm IV given within 24–48 h of delivery, followed by 10 gm monthly for 3–6 months. IV immunoglobulin therapy is safe to use in pregnancy. Adverse effects include headache, infusion reaction, and rare allergic reactions, as well as rare problems with CBC and liver enzymes.

#### Plasma exchange:

Plasma exchange, similar to IV immunoglobulin therapy, is not a proven treatment for MS. It has been used in two distinct situations: as treatment for acute relapses and as ongoing pulse maintenance therapy for MS. Based on limited data, approximately 40% of CNS inflammatory demyelinating attacks (including MS relapses) which are glucocorticoid unresponsive will respond to plasma exchange performed within 6 weeks of the attack onset.[72] A recent small uncontrolled series reported an even higher response.[73] MS relapses which involve antibody- and complement-mediated damage (so-called pattern II) often associated with MRI findings of ring enhancement, or a hypointense rim around T2 hyperintense lesions, were also reported to respond to plasma exchange within 3 days.[72] A small (*n* = 54) randomized, controlled, double-blind study in progressive MS reported a benefit from regular plasma exchange after 5 months.[72] These patients were all on cyclophosphamide plus methylprednisolone. Pulse plasma exchange should only be used for patients who show a convincing benefit.

### The Future

There have been tremendous advances in the treatment of MS over a relatively short period. Nevertheless, there is still room for improvement, and there are still unmet needs [Table 6]. First, no effective disease-modifying agents are established for use in progressive forms of MS. Second, none of the current agents are cures. They all require parenteral administration and, particularly over time, injection issues become an increasing problem in many patients. Although there are new oral agents on the horizon that appear to show even greater efficacy, the major issue with these agents will be their short- and long-term safety/side effect profiles. Third, the issue of using strong immune suppressive therapy early, to quickly control the damage process and perhaps improve long-term outcome, is an attractive concept. The issue is whether it offers true benefit, and how to select the high-risk subpopulation who would qualify for such induction therapy. The issue of combination therapy for MS is also attractive because there are so many damage mechanisms in this disease, but there is a need for formal trials to document a synergistic effect. The financial costs of combining expensive agents are likely to become an increasing concern as well. Fourthly, there is an urgent need for biomarkers to predict response to given disease-modifying agents based on class, as well as to determine response after patients have been on treatment for a short period of time, rather than having to wait a year or more to determine suboptimal responders/treatment failures. Such biomarkers may be sets of genes that are upregulated or downregulated or there may be immune responses that are produced by MS responders *vs* nonresponders. A great deal of research is being done in this area.

**Table 6:** Future issues to improve MS therapy

Develop proven therapies for the neurodegenerative (progressive) phase Develop more effective and convenient agents that are still well tolerated Determine the appropriate role for induction therapy and combination therapy Develop biomarkers -to predict therapeutic response based on class of agent -to determine therapeutic response early Establish CNS repair strategies

Finally, meaningful CNS repair strategies are needed. None of the disease-modifying agents reverses fixed damage. This therapeutic approach is needed for more disabled patients, including those with progressive MS. Such repair strategies are likely to be invaluable for multiple CNS destructive disorders in addition to MS.
